# Genetic analysis of male sterility obtained from a rice cultivar Lebed backcrossed with Taichung 65

**DOI:** 10.1186/s12284-018-0222-5

**Published:** 2018-05-03

**Authors:** Tetsuya Murakami, Tomohiko Kazama, Kinya Toriyama

**Affiliations:** Graduate School of Agricultural Science, Tohoku University, Sendai, 980-8572 Japan

**Keywords:** Male sterility, *Oryza sativa*, Sporophytic pollen killer

## Abstract

**Background:**

Male sterility is a useful agronomic trait for breeding of self-pollinating crops and is often observed in the progenies of hybrids of distantly related species, for example, *Oryza sativa* L. subsp. *indica* and *O. sativa* L. subsp. *japonica*. To explore new male sterile lines in rice, we performed successive backcrosses using a *japonica* cultivar Taichung 65 (T65) as a recurrent pollen parent and various *indica* cultivars as the initial female parents.

**Findings:**

We observed male sterile plants in the backcross progeny from an *indica* cultivar, Lebed. Both fertile and sterile plants were present in the BC_4_F_1_ generation. The sterile plants segregated for fertile and sterile plants when backcrossed with T65 in BC_5_F_1_, BC_6_F_1_ and BC_7_F_1_ with a ratio of 1:1. Conversely, all the backcross progenies from the fertile BC_4_F_1_ were consistently fertile. Anthers of the male sterile line were stunted and did not shed pollen; cross-sectional observations revealed defects in sporophytic cells. The male sterility appears to be caused by heterozygous alleles derived from T65 and Lebed. A male sterility gene was mapped between two INDEL markers on the long arm of chromosome 10, which corresponded to a 407 kb region in the Nipponbare genome.

**Conclusions:**

Since the heterozygous Lebed allele acts as dominant sporophytic pollen killer, it would be useful for recurrent selection breeding of *japonica* rice.

**Electronic supplementary material:**

The online version of this article (10.1186/s12284-018-0222-5) contains supplementary material, which is available to authorized users.

## Findings

Male sterility is a useful agronomic trait for the production of F_1_ hybrids in self-pollinating crops, eliminating laborious emasculation (Huang et al. 2014). Male sterility is often observed in the progenies of hybrids of distantly related species, for example, hybrids of *indica* group rice (*Oryza sativa* L. ssp. *indica*) and *japonica* group rice (*O. sativa* L. ssp. *japonica*). Such inter-subspecific hybrid male sterility is thought to be controlled by interaction between *indica* alleles and *japonica* alleles of several loci such as *Sa, Sb, Sc, Sd,* and *Se* (Ouyang et al. 2010). Male sterility can also be caused by incompatibility between mitochondrial and nuclear genomes in nuclear/cytoplasm substitution lines (Huang et al. 2014), referred to as cytoplasmic male sterility.

To explore new cytoplasmic male sterile lines in rice, we produced many cytoplasm substitution lines of a *japonica* cultivar Taichung 65 (T65) by successive backcross, with various rice cultivars as the initial female parent and T65 as a recurrent pollen parent. During this process, we found a male sterile line in the backcross progeny from an *indica* cultivar, Lebed, from the Philippines, which was provided by the National Institute of Agrobiological Sciences Genebank (Tsukuba, Japan) as WRC23 (Kojima et al. 2005).

An F_1_ hybrid between Lebed and T65 was backcrossed four times with T65. The resulting BC_4_F_1_ generation contained two sterile plants and four fertile plants. The progeny of a fertile plant backcrossed with T65 were all fertile (Additional file 1: Table S1). In contrast, segregation of fertile and sterile plants was observed in the BC_5_F_1_ generation obtained from a sterile plant backcrossed with T65. Seed setting rates of each plant indicated that four plants were fertile, while six plants were sterile (Additional file 1: Table S2). When the sterile line was backcrossed with T65, sterile and fertile plants were always segregated in BC_5_F_1,_ BC_6_F_1,_ and BC_7_F_1_ generations, with an overall ratio of 18:14, corresponding to a theoretical 1:1 ratio (χ^2^ = 0.5, *p* > 0.05). This indicates that the male sterility was caused by heterozygous alleles derived from T65 and Lebed in a single locus. We named the male sterility gene *LEBED-TAICHUNG 65 MALE STERILITY (LTMS)*, and designated the sterile plant as the LTMS line.

**Table 1:** Chromosomal position of markers, and genotypes, anther phenotypes, and seed setting rates in BC_6_F_1_ recombinant plants

Plant No.*		(5)- 55	(3)-55	(5)-17	(6)-28	(6)-19	(2)-28
Seed setting rate (%)		0	15.4	2.9	0.6	70.2	54.7
Anther phenotype**		I	I	I	I	D	D
Marker	IRGSP-1.0 position (kbp)						
KNJ8-indel702	11,263	T	T	T	T	T	T
KNJ8-indel704	11,269	T	T	T	T	T	T
KNJ8-indel707	11,410	H	H	H	H	T	T
KNJ8-indel709	11,609	H	H	H	H	T	T
KNJ8-indel710	11,624	H	H	H	H	T	T
C5-indel8296	11,676	T	H	H	H	H	T
C5-indel8297	11,742	T	H	H	H	H	T
C5-indel8299	11,862	T	T	H	H	H	H
C5-indel8300	11,873	T	T	H	H	H	H
KNJ8-indel711	11,882	T	T	T	H	H	H
KNJ8-indel713	11,950	T	T	T	T	H	H
KNJ8-indel714	11,954	T	T	T	T	H	H

H, Lebed/T65 heterozygous genotypes; T, T65 homozygous genotypes.

*Plant No. of BC_5_F_1_ shown in parentheses.

**Fig. 1:**
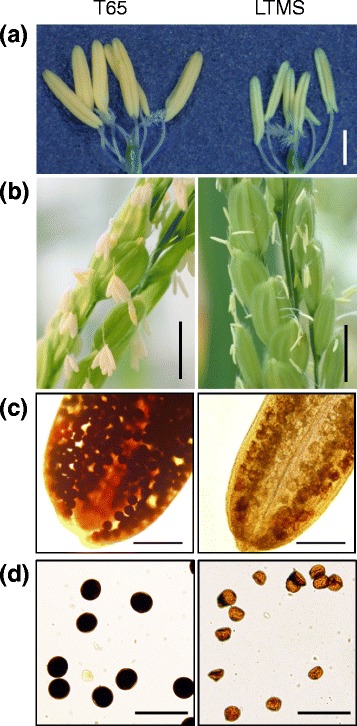
Anthers and pollen of the LTMS line and Taichung 65 (T65). (**a**) Anthers before flowering (Bar = 1 mm). (**b**) Anthers after flowering (Bar = 5 mm). (**c**) Pollen in anthers stained with I_2_-KI (Bar = 200 μm). (**d**) Pollen grains stained with I_2_-KI (Bar = 100 μm)

**Anther phenotype was judged from predominant appearance of dehiscent and indehiscent anthers at flowering stage as shown in Fig. 1b. D, dehiscence; I, indehiscence

A day before flowering, anthers were whitish yellow and stunted in the LTMS line, while those of T65 were yellow and engorged (Fig. 1a). Fig. 1b shows anthers protruding from florets just after flowering. No pollen grains were shed from anthers of the LTMS line after flowering. In contrast, empty anthers, following the shedding of pollen grains, were evident in T65 (Fig. 1b). These phenotypes of anthers, indehiscence and dehiscence, were used to identify sterile and fertile plants, respectively, for the mapping of the *LTM*S gene. Microscopic observation of anthers and pollen grains revealed that pollen grains were not stained with 0.1% potassium iodide in the LTMS line, while they were darkly stained in T65, indicating no starch accumulation in the pollen grains of the LTMS line (Fig. 1c and d). The anthers of F_1_ hybrids between Lebed and T65 contained both starch-engorged and empty pollen grains, showing a seed setting rate of 36.2%, as against a 51.3% seed setting rate in Lebed (Additional file 2: Figure S1). Relatively higher seed setting rate and pollen stainability of the F_1_ plant (Additional file 2: Figure S1), compared with those of the LTMS line, suggests that Lebed and the F_1_ plant might carry an epistatic restorer gene to suppress the function of the LTMS gene. It is interesting to note that the LTMS phenotype was only manifested in progenies backcrossed with T65.

**Fig. 2:**
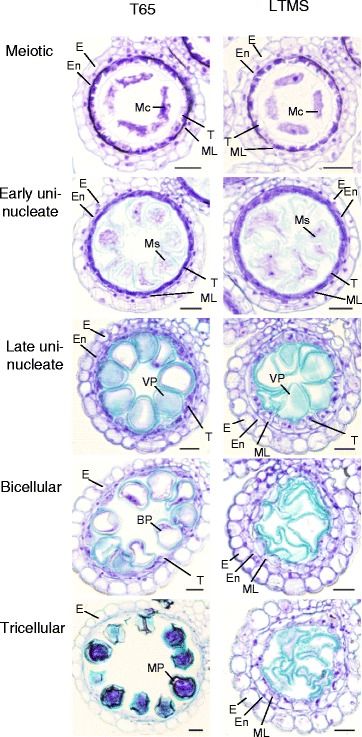
Transverse section of anthers at meiotic, early and late uninucleate microspore, bicellular pollen, and tricellular pollen stages in the LTMS line and Taichung 65 (T65). BP, bicellular pollen; E, epidermis; En, endothecium; Mc, meiocyte; ML, middle layer; MP, mature pollen; Ms., microspore; T, tapetal cell; VP, vacuolated pollen. Bar = 20 μm

Anthers at the meiotic stage, the early and late uninucleate microspore stages, the bicellular pollen stage, and the tricellular pollen stage were fixed with a formaldehyde-acetic acid-ethanol solution, and embedded in Technovit 7100 resin (Kluzer, Wehrheim, Germany) as described by Zhou et al. (2011). Transverse sections (7 μm) were stained with 0.05% toluidine blue (Fig. 2). In anthers of T65, microspores developed into pollen grains full of starch. In contrast, pollen grains were empty in the LTMS lines. Morphological differences between T65 and the LTMS line were not evident until the early uninucleate microspore stage, and development ceased at the late uninucleate microspore stage in the LTMS line. Observation of anther walls revealed that the tapetum began to degenerate in T65 at the late uninucleate microspore stage, whereas tapetal, middle layer, and endocecium cells were clearly observed in the LTMS line. The middle layer and endocecium cells of the LTMS line were enlarged at the tricellular pollen stage, whereas they were degraded in T65. These results indicate that defects in sporophytic cells cause pollen abortion.

The genome of the LTMS line in the BC_5_F_1_ generation was expected to consist predominantly of the T65 genome, except for a segment containing the *LTMS* gene. To reveal any genomic regions derived from Lebed, genotypes were determined for 65 INDEL markers covering all 12 chromosomes (Additional file 1: Table S3; Yonemaru et al. 2015). Genotyping of 10 plants revealed that a heterozygous Lebed /T65 genotype was detected only at INDEL759 in chromosome 10, as shown in Additional file 1: Table S4, while other parts were substituted with T65 chromosomal segments. This result indicates that the *LTMS* gene exists near INDEL759 in chromosome 10. Genotyping was further continued using newly designed INDEL and SSR markers (Additional file 1: Table S3). The sterile phenotype was consistent with the presence of a heterozygous Lebed /T65 genotype at each DNA marker locus between KNJ8-indel710 and SSRi98, while all the fertile plants showed T65 homozygous genotypes (Additional file 1: Table S5). These results indicate that the *LTMS* gene is located between KNJ8-indel702 (IRGSP-1.0 position: 11.26 Mb) and KNJ8-indel756 (IRGSP-1.0 position: 15.04 Mb), corresponding to a 3.7-Mb region in the Nipponbare genome (International Rice Genome Sequencing Project, IRGSP-1.0; Kawahara et al. 2013; Sakai et al. 2013).

Fine scale mapping was carried out using 384 BC_6_F_1_ and 192 BC_7_F_1_ plants. Genotyping of markers within the candidate regions (KNJ8-indel710 and KNJ8-indel756) identified 55 recombinant plants. Additional file 1: Table S6 shows the recombinant genotypes between KNJ8-indel710 and KNJ8-indel756, as well as anther phenotypes and seed setting rates, which were used to identify fertile and sterile plants. Thirty sterile plants carried heterozygous Lebed /T65 genotypes at the KNJ8-indel710 locus (Additional file 1: Table S6), while 25 fertile plants carried T65 homozygous genotypes. Some sterile plants in Additional file 1: Table S6 showed variable seed setting rates in panicles. We also noticed that some panicles of the LTMS lines occasionally set a few seeds (Additional file 1: Table S2). We do not know the reason of this occasional recovery of seed setting. Six recombinant plants were further investigated using additional INDEL markers. Four plants carrying heterozygous Lebed /T65 genotypes at KNJ8-indel707 and KNJ8-indel710 were shown to be male sterile (Table 1). We conclude that the *LTMS* gene is located between KNJ8-indel704 (IRGSP-1.0 position: 11,269 kb) and C5-indel8296 (IRGSP-1.0 position: 11,676 kb) in chromosome 10, corresponding to a 407 kb-region in the Nipponbare genome.

Based on the integrated rice science database, Oryzabase, only a single gene is listed as a hybrid sterility gene on chromosome 10—*F1 POLLEN STERILITY 18* (*S18*). The *S18* locus has been reported in backcross progenies of *japonica* (*Oryza sativa* L. ‘T65’) and African rice (*O. glaberrima* Steud., Acc. IRG104038) as a new locus affecting F_1_ pollen sterility, and shown to be linked to RFLP markers G1084 and R1629 (Doi et al. 1998). The map position corresponds to a Nipponbare genomic region between IRGSP-1.0 position 10,637 kb and 12,757 kb, which includes the candidate region of the *LTMS* gene. It is possible that the *LTMS* gene is identical to *S18*, although the originated species are different; the *LTMS* gene from *indica* rice (*O. sativa*) and the *S18* gene from African rice (*O. glaberrima*). Efforts are now in progress for molecular cloning of the *LTMS* gene.

Several hybrid sterility genes/QTL have been reported in hybrids between *O. sativa* subsp. *japonica* and subsp. *indica*. For F_1_ pollen sterility; for example, *Sa* in chromosome 1, *Sb* (*S24*) in chromosome 5, *Sc* in chromosome 3, *Sd* (*S35*) in chromosome 1, and *Se* (*S25*) in chromosome 12 were identified through a series of allelic testcrosses (Guo et al. 2016). Pollen grains carrying *japonica* alleles of *Sa*, *Sb*, *Sc*, *Sd* or *Se* are known to cause selective pollen abortion in F_1_ heterozygous plants, resulting in the predominant transmission of *indica* alleles to their progeny. Molecular cloning has been reported for the *Sa* and *Sc* genes (Long et al. 2008, Shen et al. 2017). The *Sa* locus comprises two adjacent genes, *SaM* and *SaF*, encoding a small ubiquitin-like modifier E3 ligase-like protein and an F-box protein, respectively (Long et al. 2008). Male semi-sterility occurred only when heterozygous *SaM* alleles (*SaM*^*+*^*/SaM*^*−*^) and at least one *SaF* allele (*SaF*^*+*^*/Saf*^*−*^ *or SaF*^*+*^*/SaF*^*+*^) were present, causing pollen abortion only in pollen grains carrying the *SaM*^*−*^ allele. It is possible that the *LTMS* locus is also composed of several adjacent genes. Shen et al. (2017) reported that the *japonica* allele of the *Sc* gene (*Sc-j*) corresponds to *Os03g0247300*, which encodes a 446 amino-acid protein with a DUF1618 domain. *Sc-j* has been shown to be essential for pollen development. It has been reported that *Sc-i* contains two or three tandem-duplicated *Sc-j*-homologs with distinct promoters, and the high expression of *Sc-i* in *Sc-j/Sc-i* hybrids causes suppression of *Sc-j* expression in pollen and selective abortion of *Sc-j*-pollen (Shen et al. 2017). The gene dosage-dependent allelic suppression has been proposed as a mechanism of hybrid sterility.

Hybrid pollen sterility is caused by the reciprocal gene loss of duplicated genes has also been reported. In the F_1_ hybrid between a *japonica* cultivar, Nipponbare, and an *indica* cultivar, Kasalath, pollen sterility is caused by interaction between *DOPPELGANGER1* (*DPL1*; *LOC_Os01g15448*) and *DOPPELGANGER2* (*DPL2*; *LOC_Os06g08510*). The Kasalath allele of *DPL2* and the Nipponbare allele of *DPL1* have been reported to be non-functional, and pollen carrying the two defective *DPL* alleles fails to germinate (Mizuta et al. 2010). Molecular cloning of another hybrid sterility gene has been reported in F_1_ hybrids between *O. sativa* ssp. *japonica* and *O. glumaepatula*. Hybrid sterility is governed by interaction of the *S28* gene on chromosome 4 and the *S27* gene on chromosome 8. The reciprocal loss of the duplicated genes encoding mitochondrial ribosomal protein L27 has been reported to cause pollen sterility (Yamagata et al. 2010). Notably, the above-mentioned pollen sterility genes act as gametophytic pollen killers, whilst the *LTMS* gene identified in our study acts as a sporophytic pollen killer.

Although most genic male sterility is caused by loss-of-function alleles of genes essential for anther and pollen development (see Wang et al. 2013 for reviews), two dominant genic male sterile mutants have also been reported in rice. The Pingxiang dominant male-sterile gene was designated as *Ms-p* and mapped on the SSR markers RM311 and RM3152 on chromosome 10 (Huang et al. 2007). We noticed that the mapping position of *Ms-p* corresponds to a Nipponbare genomic region between IRGSP-1.0 position 19,120 kb to 19,416 kb, which is near, but different from, the position of the *LTMS* gene in our study (Table 1). Molecular cloning of the *Ms-p* gene has not yet been reported. The gene for Sanming dominant male-sterility was named *SMS* and mapped on chromosome 8 (Pang et al. 2017). The SMS dominant male sterile line has been effectively used for recurrent selection breeding to obtain multiple abiotic stress tolerant rice cultivars (Pang et al. 2017).

Although we do not know if the male sterility at the *LTMS* locus is due to dominant genic male sterility caused by the Lebed allele alone or *indica-japonica* hybrid male sterility caused by interaction of the Lebed and T65 alleles*,* our study demonstrates that the heterozygous Lebed allele of the *LTMS* gene acts as a dominant sporophytic pollen killer in a nuclear background of *japonica* T65. The LTMS line, therefore, can be used as a dominant male sterile line in recurrent selection breeding for facilitation of population improvement of *japonica* rice.

## Additional files

Additional file 1: **Table S1.** Seed setting rates of BC_5_F_1_ plants obtained from fertile BC_4_F_1_ plants. **Table S2.** Seed setting rates of BC_5_F_1_ plants obtained from sterile BC_4_F_1_ plants. **Table S3.** Primer sequences of INDEL and SSR markers. **Table S4.** Genotyping of INDEL markers in BC_5_F_1_ plants segregating for fertility. **Table S5.** Genotyping of INDEL and SSR markers in sterile and fertile BC_6_F_1_ plants. **Table S6.** Genotypes of INDEL and SSR markers, anther phenotypes and seed setting rates in BC_6_F_1_ and BC_7_F_1_ recombinant plants. (XLSX 40 kb)

Additional file 2: **Figure S1.** Pollen grains, seed setting rates and pollen stainability of F_1_ hybrids between Lebed and T65, compared with Lebed and the LTMS line. Pollen grains were stained with I_2_-KI. (Bar = 100 μm). (PPTX 889 kb)

## Abbreviations

LTMS: LEBED-TAICHUNG 65 MALE STERILITY
T65: Taichung 65
