# Supplementary material for: Genetic analysis of male sterility obtained from a rice cultivar Lebed backcrossed with Taichung 65
Source: Rice (N Y). 2018 May 3;11:30. doi: 10.1186/s12284-018-0222-5 (PMC5934291; doi:10.1186/s12284-018-0222-5)
Supplement: Supplementary file 2 — Figure S1. Pollen grains, seed setting rates and pollen stainability of F1 hybrids between Lebed and T65, compared with Lebed and the LTMS line. Pollen grains were stained with I2-KI. (Bar = 100 μm). (PPTX 889 kb) [file 12284_2018_222_MOESM2_ESM.pptx]

## Slide 1
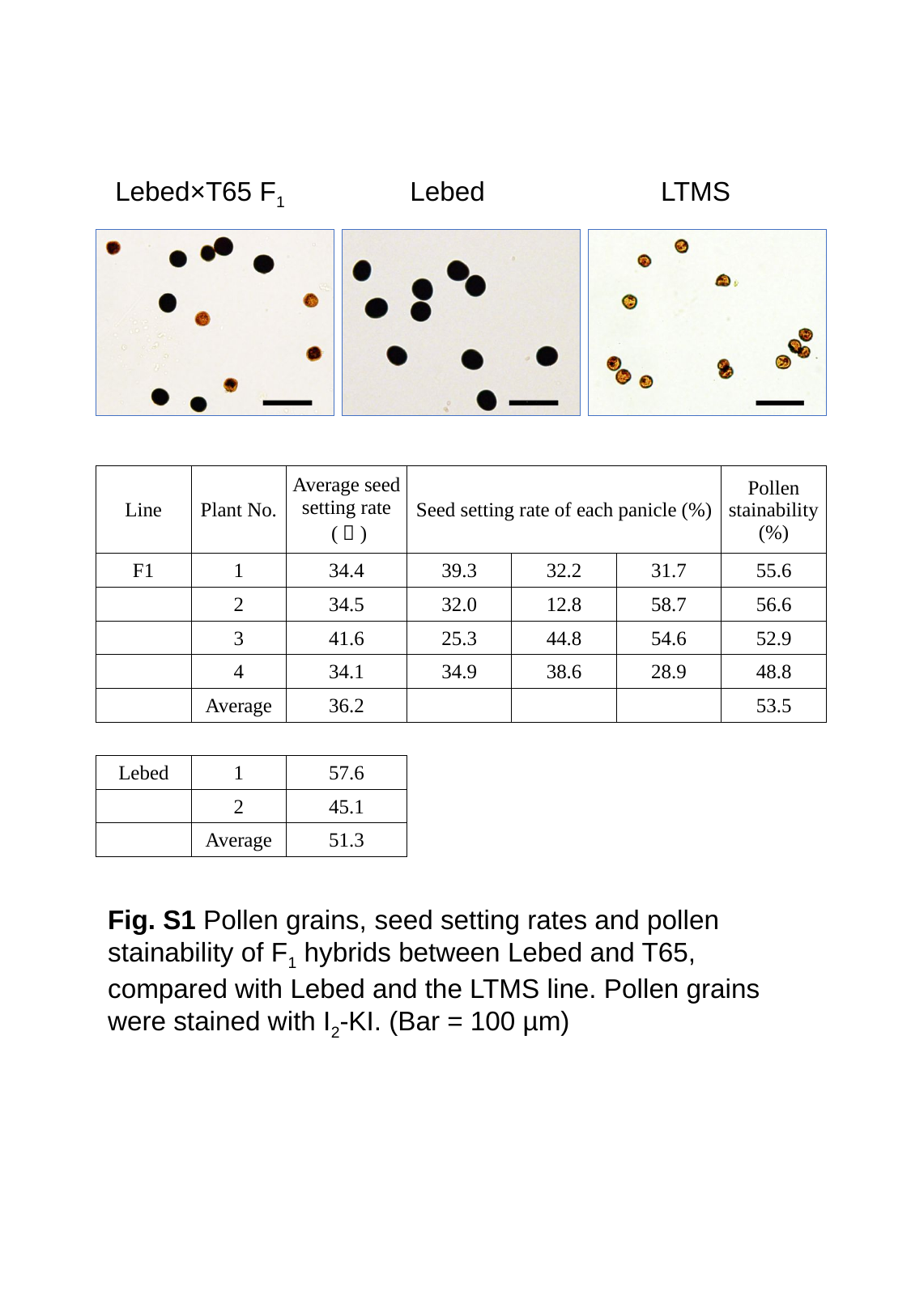

Lebed×T65 F1
Lebed
LTMS
| | | | | | | |
| --- | --- | --- | --- | --- | --- | --- |
| Line | Plant No. | Average seed setting rate (％) | Seed setting rate of each panicle (%) | | | Pollen stainability (%) |
| F1 | 1 | 34.4 | 39.3 | 32.2 | 31.7 | 55.6 |
| | 2 | 34.5 | 32.0 | 12.8 | 58.7 | 56.6 |
| | 3 | 41.6 | 25.3 | 44.8 | 54.6 | 52.9 |
| | 4 | 34.1 | 34.9 | 38.6 | 28.9 | 48.8 |
| | Average | 36.2 | | | | 53.5 |
| | | | | | | |
| Lebed | 1 | 57.6 | | | | |
| | 2 | 45.1 | | | | |
| | Average | 51.3 | | | | |
Fig. S1 Pollen grains, seed setting rates and pollen stainability of F1 hybrids between Lebed and T65, compared with Lebed and the LTMS line. Pollen grains were stained with I2-KI. (Bar = 100 µm)
